# Studying the movement behavior of benthic macroinvertebrates with automated video tracking

**DOI:** 10.1002/ece3.1425

**Published:** 2015-03-17

**Authors:** Jacqueline Augusiak, Paul J Van den Brink

**Affiliations:** 1 Aquatic Ecology and Water Quality Management Group, Wageningen University and Research centre, Wageningen UniversityP.O. Box 47, 6700 AA, Wageningen, The Netherlands; 2 Alterra, Wageningen University and Research centreP.O. Box 47, 6700 AA, Wageningen, The Netherlands

**Keywords:** Aquatic macroinvertebrates, crustaceans, dispersal, locomotion, marking

## Abstract

Quantifying and understanding movement is critical for a wide range of questions in basic and applied ecology. Movement ecology is also fostered by technological advances that allow automated tracking for a wide range of animal species. However, for aquatic macroinvertebrates, such detailed methods do not yet exist. We developed a video tracking method for two different species of benthic macroinvertebrates, the crawling isopod *Asellus aquaticus* and the swimming fresh water amphipod *Gammarus pulex*. We tested the effects of different light sources and marking techniques on their movement behavior to establish the possibilities and limitations of the experimental protocol and to ensure that the basic handling of test specimens would not bias conclusions drawn from movement path analyses. To demonstrate the versatility of our method, we studied the influence of varying population densities on different movement parameters related to resting behavior, directionality, and step lengths. We found that our method allows studying species with different modes of dispersal and under different conditions. For example, we found that gammarids spend more time moving at higher population densities, while asellids rest more under similar conditions. At the same time, in response to higher densities, gammarids mostly decreased average step lengths, whereas asellids did not. Gammarids, however, were also more sensitive to general handling and marking than asellids. Our protocol for marking and video tracking can be easily adopted for other species of aquatic macroinvertebrates or testing conditions, for example, presence or absence of food sources, shelter, or predator cues. Nevertheless, limitations with regard to the marking protocol, material, and a species’ physical build need to be considered and tested before a wider application, particularly for swimming species. Data obtained with this approach can deepen the understanding of population dynamics on larger spatial scales and of the effects of different management strategies on a species’ dispersal potential.

## Introduction

Movement ecology has received increasing attention over the years with technological advancements yielding ever more precise location devices to gain a better understanding of what influences the movement and distribution of animals (Nathan et al. 2008; Schick et al. 2008). So far, studies of movement behavior focused mostly on larger animals living in environments where their movement can be followed rather easily. Examples range from observations of migrating birds, to wandering whales, to mice, and other rodents (e.g., Edwards et al. 2007; Gurarie et al. 2009; Humphries et al. 2012). With improving technology, the number of studies on smaller species has increased, whereby terrestrial examples such as collembolans and ants are frequently chosen as study objects (Amorim et al. 2008; Robinson et al. 2008). Aquatic invertebrates and their population distributions, however, are mostly studied in time and labor intensive field surveys where a defined area is chosen and the occurring species quantified (Céréghino et al. 2001; Malmqvist 2002). Mark and recapture studies (e.g., Davy-Bowker 2002) are used as a variation of this method. Despite improving insights into dispersal times and patterns, they can over- or underestimate realized dispersal by overlooking patch-specific effects on individual behavior (Ovaskainen 2004; Van Dyck and Baguette 2005). Hawkes (2009) reviewed studies that aimed to link dispersal and population processes to investigate different ways in which they can be combined to yield an understanding of spatial population distributions. He found that the resulting metapopulation models were sensitive to small differences in the dispersal estimates. Consequently, he proposes that in order to estimate dispersal more realistically, individual variability of behavior should be accounted for.

Long-distance dispersal can be estimated from the small-scale behavior of a species (Turchin 1998). Respective studies, for example, in the laboratory via video tracking, make it possible to investigate mechanistic drivers of movement behavior. This facilitates the estimation of dispersal distances under various conditions with reduced efforts compared to field surveys. Currently, the behavior of small organisms is typically recorded via cameras installed above an arena, and the obtained paths are analyzed with computer software (Martin 2004). Often, the observed individuals are marked. However, choices concerning marking protocols depend strongly on the research question as well as detection requirements of the applied tracking software and the animals’ capability to cope with a marker and the marking procedure (Hagler and Jackson 2001).

Compared to terrestrial species, additional technical challenges need to be overcome for studying aquatic macroinvertebrates. Such problems include refraction and light reflection interferences at the air/water boundary, positioning of the light source, and suitable marking techniques. Probably due to these technical challenges, so far only a few behavioral studies have been conducted for aquatic macroinvertebrates (e.g., Englund and Hambäck 2004). Holyoak et al. (2008) also found in a review that most reported studies on invertebrate movement were performed at the population level without quantifying individual variation of behavior. This limits the understanding of factors that control behavior.

Learning more about the movement of benthic macroinvertebrates is urgently needed. As consumers at the intermediate trophic level, macroinvertebrates fulfill an important role in the nutrient cycling of aquatic ecosystems (Wallace and Webster 1996). Chemical or physical disturbances due to human activities such as agricultural or engineering practices can lead to local population declines (Vaughn 2010). The immigration of unaffected, or temporary emigration of affected individuals, can support the recovery of disturbed populations (Brederveld et al. 2011; Galic et al. 2013).

**Figure 1:**
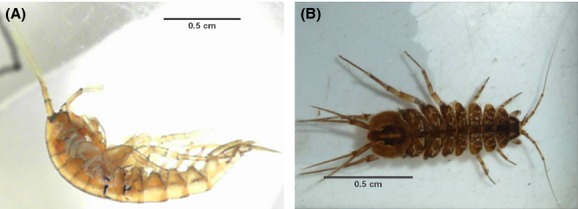
Specimens of (A) adult *Gammarus pulex* and (B) adult *Asellus aquaticus* used in the experiments.

We developed an experimental method to overcome the technical challenges described above to enable the study of movement behavior of aquatic macroinvertebrates. We tested our method with two species with different modes of dispersal, the crawling isopod *Asellus aquaticus* and the swimming freshwater amphipod *Gammarus pulex* (Fig.1). The developed method allows studying individuals of small aquatic macroinvertebrates under various test conditions, which is demonstrated in this study by varying the population densities in the test setups.

## Materials and Methods

### Test organisms

*Gammarus pulex* is an amphipod species that disperses over short distances by swimming, whereby *Asellus aquaticus* is an isopod that moves along the benthos by crawling. Both species are widely spread throughout freshwater habitats in Europe. Despite their different dispersal modes, the predominant dispersal plane is 2-dimensional for both species.

Adult *A. aquaticus* and *G. pulex* were collected during springtime in 2011 and 2012, respectively, from a noncontaminated pond (Duno pond, Doorwerth, the Netherlands) using sweeping nets. To obtain a narrow body size range, specimens of *A. aquaticus* that were larger than approximately 0.5 cm and *G. pulex* larger than approximately 1 cm were transferred to the laboratory and kept in separate, aerated 30 L tanks in a climate-controlled room at 20°C and a 10:14 light–dark cycle. Prior to the experiments, the organisms were acclimated to copper-free water in a sequential diluting process of the original pond water with copper-free water during 1 week. Dried poplar leaves were supplied as food source ad libitum.

### Experimental setup

The movement observations were performed in a climate-controlled room at 20°C. The test setup consisted of a digital single-lens reflex camera (EOS 1100D, Canon) mounted above an aquarium of approximately 1 m², which was filled with a 0.5-cm layer of quartz sand and 10 cm of copper-free tap water. The camera was directly connected to a computer. Four of such aquarium-camera combinations were installed and used in parallel.

Before the observations, individuals for the experiments were randomly chosen from the stock (mean size *A. aquaticus*: 6.4 ± 0.66 mm; mean size *G. pulex*: 13.1 ± 1.76 mm) and marked (see below). After 1 h recovering from the tagging procedure, they were introduced to the aquarium. After another 30 min for acclimation, animal movements were recorded for 1 h and the tracks analyzed. All experimental trials were replicated twenty times with different individuals. For those setups designed to investigate the influence of population densities, only one individual of the group was marked and observed, while the unmarked ones served as “background” population. When the recording was finished, the marked individual was exchanged for another marked one. The background population was exchanged after 4 h to prevent potential starvation induced behavioral changes, such as, in the case of *Gammarus*, cannibalistic tendencies.

Water temperature, pH, and dissolved oxygen were measured twice a day. All experiments were carried out during daytime in a dark room. The average water temperature was 20 ± 0.8°C, average pH was 7.6 ± 0.3 (pH323; WTW, Weilheim, Germany), and average dissolved oxygen levels varied around 8.6 ± 0.3 mg/L (Oxi330 with CellOx 325 sensor; WTW, Weilheim, Germany).

### Tagging procedure and marker choice

For the tagging procedure, individual animals were removed from the water, placed in a Petri dish, and their backs carefully dried with a lint-free tissue. Rectangular pieces of a fluorescent material (approx. 2 × 2 mm) were then fixed with a small amount of cyanoacrylate (Pattex, Gold Gel) to the back of the selected individuals and the animals returned into fresh water. The time limit for animals to be out of the water was set to 2 min to avoid over-stressing the marked individuals.

The employed marking material had to fulfill requirements related to size, weight, and toxicity to ensure that it would not influence the animals mechanically or by chemical release. A strong fluorescence under UV light and easiness to handle during preparation and marking were especially important. We found in preliminary experiments (see Data S1) that regular printing paper was suitable for *Asellus*, while neon colored rubber-like plastic met the requirements best for *Gammarus* (UV Gear, Mark SG Enterprises, Surrey, UK; www.uvgear.co.uk).

### Movement behavior studies

#### Tagging induced effects

**Figure 2:**
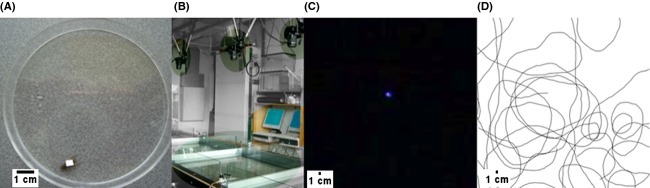
Marked *Asellus* specimen (A), the experimental setup (B), the resulting observation under UV light illumination (C), and extracted path representation (D).

To estimate potential influences of the tagging procedure and marker choice on movement behavior, we recorded marked and unmarked organisms under white light conditions. We used full-spectrum light tubes (JBL, Solar Tropic T8) as light sources, which in combination with the quartz sand substrate enabled the observation of either marked or unmarked specimens. The tubes were adjusted in positions that allowed approximately even illumination of the arenas with as little light reflection on the water surface as possible. In our case, the best positions for the light tubes were slightly to the left and right of the aquaria (Fig.2B) at a height halfway between water surface and camera, yielding an average light intensity of 2.0 ± 0.7 *μ*mol/s per m^2^ (LI-250A Light Meter; LI-COR Biosciences, Lincoln, NE). Due to limitations with extracting movement paths of multiple individuals from the movies, only single animals were introduced to the tanks and recorded. Both treatments, tagged and untagged, were alternated randomly.

#### Light induced effects

Gammarids and asellids are generally more active under dark than under light conditions (Wallace et al. 1975; Andrikovics 1981). We tested different lighting conditions to investigate light mediated differences in movement behavior. For tests under dark conditions, that is, excluding the visible wavelength spectrum, the animals were tagged with a fluorescent marker (see above) and their movement recorded while UV-A light tubes were used for illumination instead of the above-mentioned full-spectrum tubes. Figure2C illustrates the observation of a marked *Asellus* under such conditions. Single specimens were introduced into the aquaria and the recorded movement data compared to the previously acquired movement data of marked specimens under full-spectrum light conditions.

#### UV light and population density effects

We used UV light and fluorescent markers to differentiate single individuals from a background population of unmarked specimens. This made it possible to investigate the effects of population density on the behavior of individual asellids and gammarids by introducing 0, 50, 100, and 200 unmarked animals in the aquaria along with a single marked individual. These setups were performed with 20 replicates.

### Data analysis

The open source software ImageJ (Abramoff et al. 2004) was used to process and extract animal tracks from the recorded movies. Tracks within a 10 cm margin of the arena's walls were left out to exclude bias due to fence behavior (Cant et al. 2005). One image per second was processed and resulted in a series of (*x*, *y*) coordinates of an individual at time *t*. The obtained tracks were analyzed using R software (R Core Team 2013) and the R package “adehabitat” (Calenge 2006).

**Figure 3:**
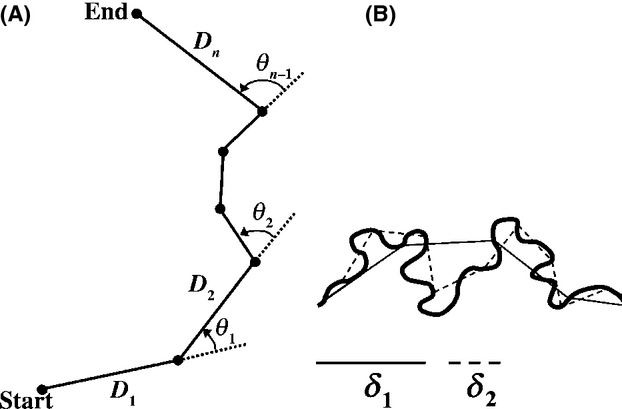
(A) Illustration of the components of a movement path. Solid lines represent the distance D_i_ travelled per time interval (step length). The dashed lines indicate the turning angle (*θ*) as the deviation from straight-line locomotion measured in degrees (±180˚). (B) Schematic of the divider method. Two steps of the analysis are shown, using two different divider lengths *δ*. (Adapted from Seuront et al. 2004)

Step length, turning angle, and overall activity are key parameters in the analysis of movement paths. Therefore, we analyzed the obtained trajectories by the distance between subsequent time points (step length); by the angle between successive moves measured as deviation from straight locomotion in degrees (±180˚); and by the time spend resting (see Fig.3A for a schematic representation of path components).

The resting times were calculated from the data as the fraction of time points when the observed individuals did not move. The smallest detectable steps were in a range of ±0.5 mm in *x* and *y* direction. We determined this value by placing paper chips used to mark *Asellus* specimen into the aquaria, recorded them for 10 min, and processed the movies like the movies with animal observations. Due to slight movements of the water phase, slight vibrations of the installed cameras or inconsistencies in camera sensor performance, the estimated *centers of gravity* of the recorded paper chips could vary by some pixels in either direction and thus caused an error of up to 0.5 mm in the position determination. Considering that both species breathe and perform other small movements when resting, we assumed that for the determination of the resting times, a larger error margin needs to be applied. We thus extended our analysis by manually choosing recording excerpts from times that we knew the animals to not move and found an error margin of up to 1 mm. Consequently, we defined steps larger than 1 mm as relocation and steps smaller than 1 mm as resting moments.

The above metrics are scale-dependent and vary depending on the physical or temporal scale at which they are measured. We used fractal analysis to analyze path tortuosity scale-independently (Seuront et al. 2004). The fractal dimension *D* of a trajectory ranges between *D *=* *1 (straight line) to *D *=* *2 (Brownian motion, eventually filling a 2-dimensional plane). We used the fractal mean estimator in the Fractal software made available by Nams (1996) to calculate the fractal dimension for each path. If multiple paths were obtained for one individual, a mean value was estimated. The software makes use of the divider method (Mandelbrot 1967) and calculates the trajectory length (*L*) over a range of divider sizes (*δ*; see Fig.3B for a schematic illustration) such that

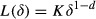
where *k* is constant and *D* the fractal dimension of the trajectory.

The fractal dimension can be calculated from a subsequent regression of log(*L*) as a function of log(*δ*). We used 200 divider sizes (*δ*) ranging from approximately half of a species’ body size (*Asellus*: 0.25 cm; *Gammarus*: 0.5 cm) to the observation scale of 100 cm.

As the fractal mean estimator excludes paths with <5 locations from the analysis to enable a robust regression result, we limited the remaining metric calculations for movement length, turning angle, and resting time to the same range to keep the data as comparable as possible. To test whether the resting time or fractal dimension (log(*D*-1)-transformed) varied among testing conditions, we used Welch's *t*-test, or in case of comparing more than two treatments, ANOVA. Because the step length data were not normally distributed, significance of differences between treatments was assessed with the Mann–Whitney U-test. The turning angles were analyzed by taking the circular nature of the data into account (Batschelet 1981; Cain 1989), that is, 180° refers to the same direction as −180°(backwards). We used a method proposed by Abuzaid et al. (2011) to represent the obtained data in form of a boxplot. For the analysis of experimental effects, data were pooled from the relocation data from all replicates for each treatment. As the distributions of the turning angles exhibited varying concentration parameters *κ* (defines how evenly distributed the data are), we used the nonparametric Watson–Wheeler test to compare treatments.

## Results

As we decided to exclude the outer 10 cm range of the aquaria from the data analysis, we did not obtain tracking information for all time points. In Table1, we list the number of data points analyzed for each testing regime along with the number of paths and their average duration. In the case of *G. pulex*, we furthermore experienced a loss of information due to the marking material. The fluorescence of the plastic markers was not as strong as the paper's. At certain angles of the swimming *Gammarus* toward the camera, the fluorescent surface was not recordable for the camera and thus also not detectable by the image processing software.

**Table 1:** Average values and standard deviations for movement parameters estimated for the different experimental regimes with *Asellus aquaticus* and *Gammarus pulex*

	1 Individual		50 Individuals		100 Individuals		200 Individuals		Light marked		Light unmarked	
	*A. aquaticus*	*G. pulex*	*A. aquaticus*	*G. pulex*	*A. aquaticus*	*G. pulex*	*A. aquaticus*	*G. pulex*	*A. aquaticus*	*G. pulex*	*A. aquaticus*	*G. pulex*
Available data points (%)	27895 (39)	1911 (3)	23563 (33)	2329 (3)	21073 (29)	3449 (5)	29511 (41)	3846 (5)	13035 (18)	12021 (17)	12162 (17)	13817 (19)
Number of available paths	328	65	375	134	321	161	408	104	172	256	157	793
Path duration (sec)	84.8 (±117.8)	26.7 (±80.1)	62.0 (±94.3)	15.0 (±19.5)	64.9 (±105.7)	19.4 (±28.2)	72.0 (±85.8)	36.0 (±60.2)	74.1 (±143.1)	46.3 (±98.9)	77.3 (±109.0)	16.3 (±19.9)
Animal activity and resting behavior												
Resting time, %	30.2 (±12.4)	39.5 (±33.7)	40.2 (±13.8)	26.0 (±28.1)	36.9 (±14.2)	20.2 (±22.9)	38.5 (±15.6)	45.7 (±18.3)	40.1 (±21.4)	47.9 (±28.6)	41.4 (±11.6)	18.2 (±18.0)
Step length pattern												
Step length (cm ±SD)	0.72 (±0.26)	1.31 (±1.47)	0.54 (±0.25)	2.14 (±2.27)	0.59 (±0.26)	2.83 (±2.25)	0.57 (±0.26)	0.67 (±0.79)	0.65 (±0.42)	1.30 (±0.92)	0.61 (±0.20)	4.13 (±1.56)
Turning behavior												
Turning angle, °	0.74 (±7.28	34.29 (±88.79)	−0.72 (±7.95)	12.28 (±67.38)	−0.07 (±7.77)	6.01 (±16.12)	−7.63 (±35.12)	−19.64 (±115.96)	0.97 (±13.0)	1.93 (±13.89)	0.07 (±6.26)	−1.8 (±6.68)
Fractal dimension *D*	1.17 (±0.13)	1.20 (±0.17)	1.10 (±0.13)	1.11 (±0.10)	1.11 (±0.12)	1.09 (±0.09)	1.11 (±0.10)	1.29 (±0.21)	1.10 (±0.11)	1.13 (±0.09)	1.10 (±0.08)	1.05 (±0.04)

### Animal activity and resting behavior

#### Effects of experimental conditions

**Figure 4:**
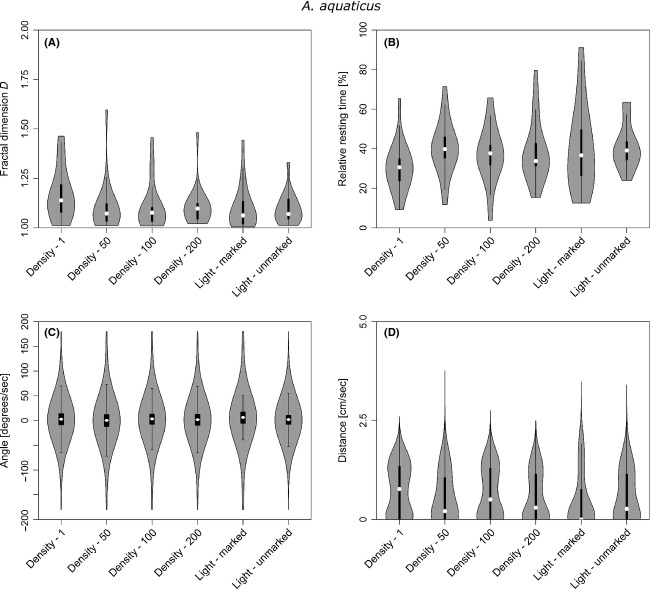
Box and Whisker plots combined with violin plots showing the effects of the different treatments on (A) the fractal dimension *D*, (B) resting times, (C) turning angles, and (D) step lengths of *Asellus aquaticus*. Violin plots are a combination of box and kernel density plots and display the probability distribution of parameters at different values (Hintze and Nelson 1998).

The marking had little influence on the average resting time of *A. aquaticus*, although the variability in resting time increased when the animals were marked (compare light unmarked with light marked in Table1 and Fig.4B). Under UV light conditions, this variability decreased and the overall distribution of resting times approached that of unmarked asellids. Furthermore, the mean resting time dropped by almost 10% under UV light conditions compared to full-spectrum light with marked test specimens (Fig.4B, Table1). Due to the relatively high variability of average resting times, this difference was not statistically significant (Table2).

**Table 2:** Summary statistics of the statistical tests to estimate the significance of the effects of experimental conditions on movement parameters from observations of *Asellus aquaticus* and *Gammarus pulex*

	Resting times^1^^,^^2^		Step length^3^^,^^4^		Turning angle^5^			Fractal dimension^1^^,^^2^^,^^6^	
	t	*P*	W	*P*	W	*P*	df	t	*P*
Marking									
*A. aquaticus*	−0.23	0.82	166	0.86	2.56	0.28	2	0.05	0.96
*G. pulex*	3.96	<0.01	29	<0.01	18.21	<0.01	2	3.57	<0.01
Light									
*A. aquaticus*	−1.69	0.11	220	0.60	3.06	0.20	2	1.81	0.08
*G. pulex*	−0.62	0.55	72	0.71	3.72	0.16	2	1.20	0.26

Density	df	F	*P*	df	Χ^2^	*P*	W	*P*	df	df	F	*P*
*A. aquaticus*	41.47	2.21	0.11	3	5.47	0.14	4.98	0.55	6	41.31	1.20	0.32
*G. pulex*	19.09	3.66	0.03	3	10.88	0.01	17.99	0.01	6	21.96	4.69	0.01

1 Welch's *t*-test for 2-sample comparison.

2 ANOVA for multisample comparison.

3 Wilcoxon's rank sum test for 2-sample comparison.

4 Kruskal–Wallis test for multisample comparison.

5 Watson–Wheeler test for 2- and multisample comparison.

6 Fractal dimension was log(*D*-1)-transformed prior to statistical testing.

**Figure 5:**
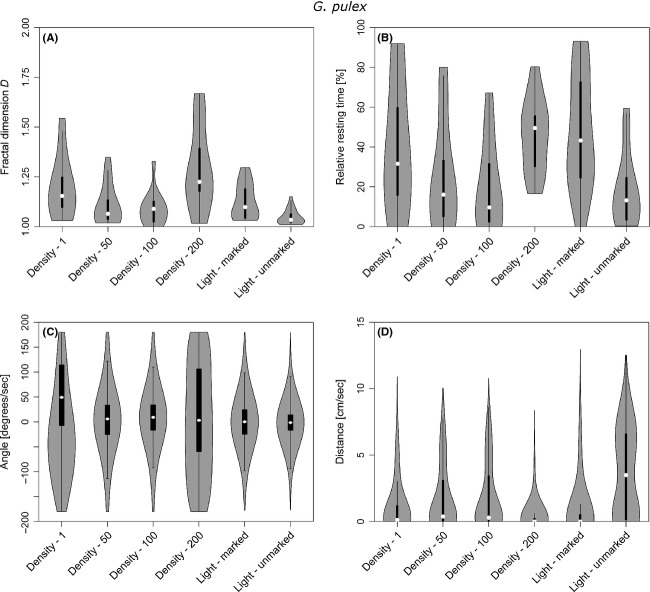
Box and Whisker plots combined with violin plots showing the effects of the different treatments on (A) the fractal dimension *D*, (B) resting times, (C) turning angles, and (D) step lengths of *Gammarus pulex*.

The resting behavior of *G. pulex*, in contrast, was significantly affected by the marking procedure (Fig.5B, Table2). The mean resting time increased drastically (Fig.5B). We also found in further analysis that the number of stops per distance increased strongly (Data S2).

#### Effects of population density

Population density did not affect the resting behavior of *A. aquaticus* significantly, which was the case for *G. pulex* (Table2). Increasing the population density of *Asellus* from one to fifty individuals per aquarium yielded the strongest change of mean resting time for that species. Further increases of *Asellus* population size returned resting times between the two testing regimes with one and fifty individuals. While the presence of unmarked individuals led to a small increase in resting time for *A. aquaticus*, the opposite occurred for *Gammarus* at densities of 50 and 100 individuals. For both species, the mentioned trends were reversed at a density of 200 individuals per m^2^ (Figs4B and 5B). Furthermore, increasing population sizes caused a small increase in variation of resting times for *AsellusAsellus*, while the opposite occurred for *Gammarus* (Table1).

### Step length patterns

#### Effects of experimental conditions

The marking procedure affected the step lengths of *AsellusAsellus* only slightly and was statistically not significant (Table2). The average step length of *AsellusAsellus* remains about the same with the marker applied, but increases when the light regime is changed from full-spectrum light to UV (Table1). The distribution of step lengths follows an exponential pattern under the full-spectrum light conditions, whereas it changes to a Lévy walk pattern where a series of small steps is interchanged with a few larger steps under dark conditions. The violin plots in Figures4D and 5D depict the distribution of data points around the boxplot representation. A Lévy walk pattern would typically be characterized by a violin with two “bulbs”, whereby the lower one would be bigger due to the presence of more short steps than large steps. An exponential distribution exhibits a broad “base bulb” with a lengthy neck.

Step lengths of *G. pulex* are significantly reduced (more than 50%) by the marking procedure (Tables1 and 2). The distribution of step lengths changed from a Lévy pattern to a more exponential one when a marker was applied (Fig.5D). We did not observe any significant changes of average step lengths when comparing light full spectrum to UV exposure although the different light sources lead to increased step lengths and a stronger Lévy pattern in the UV setup (Fig.5D).

#### Effects of population density

Changes in population density did not significantly affect the observed step lengths for *Asellus* (Tables1 and 2). The average step length was highest when the *Asellus* were alone in the arena, but remained virtually unchanged at higher densities. The form of the exhibited Lévy pattern in step length distributions also remained similar at higher densities of asellids (Fig.4D).

Step lengths of *Gammarus*, on the other hand, were significantly affected by population density (Table2). The average step lengths and their standard deviation increased up to a density of 100 gammarids/m^2^ and decreased again at the highest density (Table1), where the resting time was also clearly higher than at the two intermediate densities.

### Turning behavior

#### Effects of experimental conditions

*Asellus* hardly changed their turning behavior when marked (Fig.4C). The increase in turning angle variability due to marking and using full-spectrum light reduced the dominance of angles around 0° (forwards) not significantly (Tables1 and 2). The path tortuosity, as represented in the fractal dimension, remains almost unchanged and is only slightly wider distributed after marking. A change of the light conditions from full spectrum to UV light reversed the change of turning angle variability and lead to a distribution similar to that of unmarked conspecifics under full light spectrum conditions. The path tortuosity, however, became slightly more variable (Fig.4A).

The marking had a significant effect on the turning angle of *Gammarus* (Table2). Although the average direction remained approximately the same, the variability of angles exhibited by marked individuals was greater than of unmarked ones and the path tortuosity increased significantly as displayed in Figure5A. Changing the light regime from full spectrum to UV light also induced a strong change of the average turning angle as well as the turning angle distribution (Table1), but due to the variability of this parameter in both treatments, no statistical significance of light conditions on turning angles could be detected (Table2).

#### Effects of population density

Population density hardly affected the turning angle distribution of *A. aquaticus* (Fig.4C, Tables1 and 2). Density also had no statistically significant influence on the fractal dimension. The higher the density, however, the narrower the distribution of *D* (Table1).

As with the previous metrics, the overall directionality of gammarids was significantly affected by population density (Table2). The single gammarids performed sharper turns with an average direction that would yield less straight-line relocations. This is also observed in the fractal dimension, which has a higher distribution and average value compared to the two intermediate population densities. At the highest density level, the turning angle distribution becomes almost uniform (Fig.5C, Table1).

## Discussion

We developed a method for automated video tracking of individual, aquatic macroinvertebrates, which allows collecting detailed information about their behavior under different conditions such as varying population densities, sediment composition, light regimes, or presence/absence of other factors such as food, shelter, or stress. The presented tagging and light regime methods can also be adapted to accommodate different species with different modes of dispersal. Furthermore, the spatial and temporal scales as well as the data analysis remain flexible, which can be beneficial and important, depending on the relevant scales of either aspect for the study (Skelsey et al. 2012). The application of UV lamps and fluorescent markers proved to be a cost-efficient solution to observing aquatic macroinvertebrates while avoiding light reflections on the water surface that can interfere with the image analysis. Additionally, the differences in coloration of the study objects and the substrate, that is, sediment, are usually smaller than between the species and quartz sand that we used. In this respect, fluorescing markers can be a useful means to overcome object detection difficulties during the image processing, especially when relatively big arenas (compared to the body size of the species) are used for the experiments and only a few pixels are available to represent the animal. However, several factors require careful consideration before the method can be adopted in a meaningful way for new species.

The marking procedure affected both species, *Gammarus* more strongly than *Asellus*. However, while *Gammarus* showed effects in all analysis parameters, all of them also statistically significant, *Asellus* exhibited slightly increased variability in turning angles and path tortuosity. The crawling mode of dispersal and the lower center of gravity make asellids more stable on even grounds and thus less prone to an increase of the water resistance due to the attached markers. Any device attached to an aquatic animal will exhibit a drag which affects the animal's movement mechanics depending on the size and weight differences between device and animal. A recent study by Jones et al. (2013) illustrated that marking devices mounted on marine turtles exhibit a drag that influences energy expenditures and behavior of the turtles. In order to be visible to the camera, we had to size and position the markers on the test specimens in a way that made the markers extend slightly winglike. This may alter the hydrodynamics and thus affect the movement of *Gammarus*, especially the directionality. It was also more difficult to mark *Gammarus* individuals because they were more agile when removed from the water phase than *Asellus* and exhibited unpredictable, erratic turns. This increased the stress risk of *Gammarus* leading to a stronger impact on the overall movement behavior despite an acclimation period prior to the experiments. The mean resting time and mean number of stops made per covered distance increased along with the variability of both parameters (Figs4B and 5B, Data 2). This is most likely not only due to the physiological stress response by the (more sensitive) gammarids, but also due to the mechanical, physical impairment that the chosen material, or the way it was fixed, may have had on the swimming. Nevertheless, previous studies as the one by Freilich (1989) applied similar marking methods successfully to other macroinvertebrate species in the laboratory and in the field although the study organisms, stonefly larvae, were larger (approx. 2–5 cm) and more robust than gammarids. Also, the rubber pieces could not be designed smaller as they were not as brightly fluorescent under UV light as the paper markers and would otherwise not yield sufficient visibility. Another material choice, preferably of white color and inedible material, could overcome these problems and allow for the study of smaller or swimming species. Aiken and Roughley (1985), for example, successfully used small pieces of a plastic waterproof tape that they applied to aquatic beetles. Most other techniques of marking applicable for terrestrial invertebrates, such as powder coating or dyes, cannot be applied for aquatic invertebrates as the materials would either wash off or require dry surface tissues for fixation, which the water bound organisms may not survive. Mutilation techniques may also alter the hydrodynamics and thus affect the movement behavior already on a mechanical level. Feeding colored or fluorescent compounds, as is often carried out with microorganisms or smaller and short-lived species, carries a higher risk for intoxications of the marked organism (Hagler and Jackson 2001). Here, a possible intoxication could occur due to the use of cyanoacrylate. During the polymerization process of the glue, the surrounding water can induce a hydrolysis reaction leading to the release of small amounts of formaldehyde and alkyl cyanoacetate. A previous study, in which we tested the safety and toxicity of the chosen marking regime, however, did not indicate any severe effects on the animal's survival or behavioral endpoints (results shown in the Data S1).

Comparing the behavioral changes of both species due to marking, we would suggest that the presented marking technique would need to be refined for species that swim and/or are small, and where maintaining hydrodynamic stability thus is a bigger concern than for species that live close to the benthic area or have a flatter body design like *Asellus*.

Another factor to consider in regard to the experimental setup is the application of UV lamps. Some species of aquatic invertebrates react to this wavelength spectrum and may use it as a reference to guide diurnal or mating behavior pattern (Frank and Widder 1994). We could not find any relevant information on the photosensitivity for our particular test species and whether their retinae allow the detection of UV light. However, considering the studies of Goldsmith and Fernandez (1968) and Aarseth and Schram (1999) on spectral sensitivities of crustaceans and comparing the behavioral responses from both species when changing the light regime, we conclude that neither *Asellus* nor *Gammarus* seem to be affected by the UV range. Goldsmith and Fernandez (1968) investigated the light receptors in the eyes of different species of freshwater crustaceans and a terrestrial isopod but found only scarce occurrences of UV sensitivity for the crustaceans. Aarseth and Schram (1999) compared the vertical migration profiles of two copepod species under exposure to visible wavelengths (VIS) and a combination of VIS and UV wavelengths. They found that one species gathered deeper in the water phase when UV light was used. The other reacted only to the VIS-UV combination when they were kept in a shallow beaker closely to the light source. We did find a reduction in resting times for both species as the most notable behavioral change when using UV instead of the full-spectrum lights. Allema et al. (2012) found a similar response in terrestrial, nocturnal beetles when comparing full spectrum to red light conditions. They furthermore concluded that near infrared (NIR) light would be the most suitable to study the behavior of nocturnal organisms, but that the more practical and more readily available red light lamps would still allow a representative observation of the animals as total darkness would be rarely found in ecological environments. Goldsmith and Fernandez (1968) attributed a similar conclusion for crustaceans in general to an absence of UV wavelengths in most of the relevant aquatic habitats. The light source could be changed to NIR or red light for species that respond more strongly to UV. However, more contrast would be lost between the observed object and the background. Given the dimensions of our setup, either a stronger camera needs to be used under such circumstances or the camera would need to be lowered to increase the number of pixels representing the object of interest, which would mean that only a smaller part of the arena could be monitored.

We tested further limits of the developed protocol by studying the movement behavior of *Asellus* and *Gammarus* in different population densities. For *Asellus*, we generally found the most striking differences in behavior between the lowest densities of 1 and 50 individuals/m^2^ (Table1). Parameter values determined at higher population densities fell into ranges that were inbetween these two densities. Resting times changed the strongest. The exhibited increase in activity when alone compared to the higher densities suggests a search for conspecifics as protection mechanism against predation. A similar phenomenon was reported for the movement speed of mussels by Van de Koppel et al. (2008). They explained their findings by suggesting that an initial slowing at increasing densities was initiated by small-scale cluster formations as protection against predators. At higher densities, they found movement speeds to increase again, which was hypothesized to release intraspecific competition. Additional work by De Jager et al. (2013), furthermore, suggests that changes in movement behavior at increasing population densities can be explained by conspecific encounter rates. We find similar effects of density on both our species with an increased number of stops made per meter, reduced average step lengths, and more variable turning angles at the highest population density compared to the intermediate ones.

*Gammarus pulex* showed a different behavioral pattern at the different population densities regarding the resting time, with the biggest overall differences occurring between the intermediate densities and the 200 individuals/m^2^ experiments (Table1) and appears most active in the intermediate density ranges. This duality in inactivity, resting similarly much when alone or at higher densities, could be influenced by the marking. The presence of conspecifics seems to trigger a searching or escaping mode of behavior despite the negative influence of the markers on the hydrodynamics. Once the population density, and thus the encounter rate, become too high it might energetically be more advantageous for the marked individual to stay inactive rather than search for food or try to escape conspecifics.

Nevertheless, we rarely found statistical significance when comparing testing regimes, with the strongest indication of marking affecting the behavior of *G. pulex*. The high variability of individual behavior is a reason for this, which is amplified by the observation of 20 individuals per setup. Despite the rare statistical significances, trends in the data could possibly be magnified with appropriate methods in a modeling exercise to determine whether the small local changes in behavior yield a significant effect on a larger scale. Considering that the scale-dependent parameters exhibited patterns that are similar to the scale-independent fractal dimension indicates that our observations are representative and might not change much if a different temporal or spatial scale was applied for the analysis. In general, the data analysis, the estimation of summary statistics such as a net-squared displacement, and adjusting of the experimental environment can be designed and performed according to the respective research question. The basic experimental setup could furthermore be applied in semi-natural environments in outdoor systems if the water phase is clear enough.

To extrapolate the experimental findings to more complex scenarios or spatial scales than could be captured with a camera, modeling can be used to translate these findings from the small-scale behavior to large-scale dispersal. Models can thus help to understand how localized factors relate to dispersal events and pattern as well as the resulting distribution of populations and their connections. The experiments might only reflect a small aspect of an overall behavior on a population level in a larger, heterogeneous environment but can provide first insights into the behavioral drivers for species, which so far were not studied because of technical limitations or could be used as building blocks in mixed modeling approaches. Holdo and Roach (2013), for instance, demonstrated that Monte Carlo simulation could serve as a tool to extrapolate from small sample sizes to the population and to account for potentially different behavioral modes to capture population dispersal more realistically.

## Supporting Information

**Data S1.** Toxicological effects of markers.

**Data S2.** Additional data analysis.
