# Supplementary material for: Studying the movement behavior of benthic macroinvertebrates with automated video tracking
Source: Ecol Evol. 2015 Mar 17;5(8):1563–75. doi: 10.1002/ece3.1425 (PMC4409406; doi:10.1002/ece3.1425)
Supplement: Supplementary file 1 [file ece30005-1563-sd1.docx]

**Supporting Information 1**

***Toxicological effects of markers***

**Materials & Methods**

To test if the selected materials or the marking procedure would induce effects on mobility and mortality, tests were performed with both species (*Asellus* and *Gammarus*) and both materials (regular printing paper and UV reactive balloons obtained from UV Gear, Mark SG Enterprises, Surrey, UK; www.uvgear.co.uk). To this end, 10 individuals of either species were marked with one of the materials and kept in a 1L borosilicate beaker filled with copper-free water for 48 hours. Stainless steel hook-shaped gauze pieces were provided as structural elements and aeration was provided throughout the test duration. Unmarked individuals were treated similarly and kept under the same conditions as a control group. All treatments were set up with three replicates. Investigated parameters were immobilisation and mortality after 4, 24, and 48 hours. Criteria for defining these parameters were taken from Rubach *et al.* (2011). Individuals with affected movement compared to controls after agitation with forceps were categorised as immobile. Immobile individuals that did not react visibly within 30 seconds after repeated agitation were counted as dead.

**Results**

We found that neither marker material influenced the mobility or mortality of *A. aquaticus* in the 48 hour toxicity assessment essays. *G. pulex*, however, was more sensitive to the marking. While the plastic based markers exhibited relatively mild initial effects on mortality and mobility, the paper markers affected both endpoints more strongly during the first day (table S1). We noticed that individual Gammarids marked with paper were quickly surrounded by conspecifics, an effect that was not apparent for Asellids. A follow-up experiment revealed (results not shown) that the Gammarids perceived the paper marks as food sources and were similarly strongly attracted to them as to leaf disks. To avoid unnecessary effects of the marker material on the behaviour, we decided to use the plastic markers despite their lower fluorescence strength.

Table S1: Marker toxicity averaged over three replicates per treatment.

|  |  | **immobility** | | |  | **mortality** | | |
| --- | --- | --- | --- | --- | --- | --- | --- | --- |
|  |  | 4 h | 24 h | 48 h |  | 4 h | 24 h | 48 h |
| ***G. pulex*** | |  |  |  |  |  |  |  |
|  | **Paper** | 27% | 37% | 37% |  | 23% | 37% | 37% |
|  | **Plastic** | 10% | 20% | 40% |  | 7% | 20% | 33% |
|  | **Control** | 0% | 10% | 33% |  | 0% | 10% | 33% |
| ***A. aquaticus*** | |  |  |  |  |  |  |  |
|  | **Paper** | 0% | 0% | 0% |  | 0% | 0% | 0% |
|  | **Plastic** | 0% | 0% | 0% |  | 0% | 10% | 10% |
|  | **Control** | 0% | 0% | 0% |  | 0% | 0% | 10% |

**References**

Rubach, M.N., Crum, S.J.H. & Van den Brink, P.J. (2011). Variability in the dynamics of mortality and immobility responses of freshwater arthropods exposed to chlorpyrifos. *Archives of Environmental Contamination and Toxicology*, **60**, 708–721. Retrieved March 13, 2012, from http://www.pubmedcentral.nih.gov/articlerender.fcgi?artid=3070881&tool=pmcentrez&rendertype=abstract
