# Supplementary material for: Studying the movement behavior of benthic macroinvertebrates with automated video tracking
Source: Ecol Evol. 2015 Mar 17;5(8):1563–75. doi: 10.1002/ece3.1425 (PMC4409406; doi:10.1002/ece3.1425)
Supplement: Supplementary file 2 [file ece30005-1563-sd2.docx]

**Supporting Information 2**

***Additional data analysis***

**Materials & Methods**

From the obtained data files, the following additional parameters were extracted and analysed:

1. number of stops per walked meter (gross distance) to better understand animal activity and resting behaviour;
2. a delta-statistic to determine whether an oriented random walk or unoriented correlated random walk model would describe the movement data best (Marsh & Jones 1988; Westerberg *et al.* 2008).

The delta statistic (*Δ*) was calculated for all consecutive path sequences of length *n* and equals the difference of the mean resultant vector lengths of the global compass directions of the subsequent steps (*θ_i_*) and the turning angles between the subsequent steps (*ω_j_*):

$\Delta=\left( \frac{{(\sum{\cos\theta}_{i})}^{2}+ {(\sum{\sin\theta}_{i})}^{2}}{n^{2}} \right)-\left( \frac{{(\sum{\cos\omega}_{j})}^{2}+ {(\sum{\sin\omega}_{j})}^{2}}{{(n-1)}^{2}} \right)$.

A *Δ* –value greater than zero indicates that the distribution of compass directions is concentrated around a certain value compared, which means that the move pattern is directional. A negative *Δ*–value, on the other hand, indicates that an animal moves around without specific orientation.

To determine whether the treatments influenced the stop frequency and degree of orientation (delta-statistic) we performed Welch’s t-tests, or, in case of more than two treatments, an ANOVA.

**Results**

***Number of stops per walked distance***

The marking had little influence on the average number of stops of *A. aquaticus*, although the variability increased when the animals were marked (compare light-unmarked with light-marked in Table S2.1 and Figure S2.1a). Under UV light conditions, this variability decreased and the overall distribution of number of stops per meter approached those of unmarked Asellids (Figure 4b and S2.1a, Table S2.1). Due to the overall high variability of the average number of stops, however, this difference was not statistically significant (Table S2.2).

The resting behaviour of *G. pulex*, in contrast, was significantly affected by the marking procedure (Figure S2.1b, Table S2.2). Both, the mean resting time and the mean number of stops made per "walked" meter, increased as did the variability of both parameters (Figure S2.1b).

We did not find statistically significant effects of population density in general on the resting behaviour of *A. aquaticus* or *G. pulex* (Table S2.2). The number of stops per walked meter did not show clear trends for either species. Population density affected this parameter significantly only in the case of Gammarus (Table S2.1, Table S2.2). However, it seems that Asellus stops more frequently at higher population densities, which fits the simultaneously increased resting times. Gammarus, on the other hand, appears to stop less frequently in accordance with the reduced average resting time (Table S2.1).

***Delta statistic***

The delta statistic is negative in all cases for both species (Table S2.1) indicating that an unoriented walk model could apply to represent the collected data.

**References**

Marsh, L.M. & Jones, R.E. (1988). The form and consequences of random walk movement models. *Journal of Theoretical Biology*, **133**, 113–131. Retrieved from http://linkinghub.elsevier.com/retrieve/pii/S0022519388800286

Westerberg, L., Lindström, T., Nilsson, E. & Wennergren, U. (2008). The effect on dispersal from complex correlations in small-scale movement. *Ecological Modelling*, **213**, 263–272. Retrieved from http://linkinghub.elsevier.com/retrieve/pii/S0304380007006497

**Table S2.1:** Average values and standard deviations for movement parameters estimated for the different experimental regimes with *Asellus aquaticus* and *Gammarus pulex.*

|  | | **1 Individual** | |  | **50 Individuals** | |  | **100 Individuals** | |  | **200 Individuals** | |  | **Light - marked** | |  | **Light - unmarked** | |
| --- | --- | --- | --- | --- | --- | --- | --- | --- | --- | --- | --- | --- | --- | --- | --- | --- | --- | --- |
|  |  | *A. aquaticus* | *G. pulex* |  | *A. aquaticus* | *G. pulex* |  | *A. aquaticus* | *G. pulex* |  | *A. aquaticus* | *G. pulex* |  | *A. aquaticus* | *G. pulex* |  | *A. aquaticus* | *G. pulex* |
|  | Available data points | 27895  (39%) | 1911  (3%) |  | 23563  (33%) | 2329  (3%) |  | 21073  (29%) | 3449  (5%) |  | 29511  (41%) | 3846  (5%) |  | 13035  (18%) | 12021  (17%) |  | 12162  (17%) | 13817  (19%) |
|  | Number of available paths | 328 | 65 |  | 375 | 134 |  | 321 | 161 |  | 408 | 104 |  | 172 | 256 |  | 157 | 793 |
|  | Path length | 84.8  (± 117.8) | 26.7  (± 80.1) |  | 62.0  (± 94.3) | 15.0  (± 19.5) |  | 64.9  (± 105.7) | 19.4  (± 28.2) |  | 72.0  (± 85.8) | 36.0  (± 60.2) |  | 74.1  (± 143.1) | 46.3  (± 98.9) |  | 77.3  (± 109.0) | 16.3  (± 19.9) |
| ***Animal activity and resting behaviour*** | | | |  |  | |  |  | |  |  | |  |  | |  |  | |
|  | Resting time | 30.2 %  (± 12.4) | 39.5 %  (± 33.7) |  | 40.2 %  (± 13.8) | 26.0 %  (± 28.1) |  | 36.9 %  (± 14.2) | 20.2 %  (± 22.9) |  | 38.5 %  (± 15.6) | 45.7 %  (± 18.3) |  | 40.1 %  (± 21.4) | 47.9 %  (± 28.6) |  | 41.4 %  (± 11.6) | 18.2 %  (± 18.0) |
|  | Stops per meter | 63.2  (± 87.9) | 327.2  (± 633.1) |  | 108.3  (± 113.5) | 82.0  (± 134.8) |  | 88.5  (± 85.5) | 34.4  (± 54.8) |  | 134.5  (± 233.9) | 175.4  (± 167.5) |  | 227.8  (± 425.8) | 192.3  (± 439.4) |  | 81.6  (± 57.2) | 8.4  (± 14.2) |
| ***Step length pattern*** | | | |  |  | |  |  | |  |  | |  |  | |  |  | |
|  | Step length | 0.72 cm  (± 0.26) | 1.31 cm  (± 1.47) |  | 0.54 cm  (± 0.25) | 2.14 cm  (± 2.27) |  | 0.59 cm  (± 0.26) | 2.83cm  (± 2.25) |  | 0.57 cm  (± 0.26) | 0.67 cm  (± 0.79) |  | 0.65 cm  (± 0.42) | 1.30 cm  (± 0.92) |  | 0.61 cm  (± 0.20) | 4.13 cm  (± 1.56) |
| ***Turning behaviour*** | | | |  |  | |  |  | |  |  | |  |  | |  |  | |
|  | Turning angle | 0.74°  (± 7.28 | 34.29°  (± 88.79) |  | -0.72°  (± 7.95) | 12.28°  (± 67.38) |  | -0.07°  (± 7.77) | 6.01°  (± 16.12) |  | -7.63°  (± 35.12) | -19.64°  (± 115.96) |  | 0.97°  (± 13.0) | 1.93°  (± 13.89) |  | 0.07°  (± 6.26) | -1.8°  (±6.68) |
|  | Delta | -0.16  (± 0.14) | -0.13  (± 0.22) |  | -0.03  (± 0.07) | -0.07  (± 0.16) |  | -0.06  (± 0.15) | -0.08  (± 0.11) |  | -0.09  (± 0.09) | -0.03  (± 0.06) |  | -0.07  (± 0.09) | -0.07  (± 0.06) |  | -0.12  (± 0.08) | -0.11  (± 0.06) |
|  | Fractal dimension *D* | 1.17  (± 0.13) | 1.20  (± 0.17) |  | 1.10  (± 0.13) | 1.11  (± 0.10) |  | 1.11  (± 0.12) | 1.09  (± 0.09) |  | 1.11  (± 0.10) | 1.29  (± 0.21) |  | 1.10  (± 0.11) | 1.13  (± 0.09) |  | 1.10  (± 0.08) | 1.05  (± 0.04) |

Table S2.2: Summary statistics of the statistical tests to estimate the significance of the effects of experimental conditions on movement parameters from observations of *Asellus aquaticus* and *Gammarus pulex*.

|  |  | **Resting times ^a,b^** | | | |  | **Number of stops ^a,b^** | | | |  | **Step lengths ^c,d^** | | | |  | **Path tortuosity ^a,b^** | | | |  | **Delta index ^a,b^** | | | |  | **Turning angle ^e^** | | |
| --- | --- | --- | --- | --- | --- | --- | --- | --- | --- | --- | --- | --- | --- | --- | --- | --- | --- | --- | --- | --- | --- | --- | --- | --- | --- | --- | --- | --- | --- |
|  |  | t | | p | |  | t | | p | |  | W | | p | |  | t | | p | |  | t | | p | |  | W | p | df |
| **Marking** | | | | | | | | | | | | | | | | | | | | | | | | | | | | | |
|  | *A. aquaticus* | -0.23 | | 0.82 | |  | 1.52 | | 0.14 | |  | 166 | | 0.86 | |  | 0.59 | | 0.56 | |  | -0.31 | | 0.76 | |  | 2.56 | 0.28 | 2 |
|  | *G. pulex* | 3.96 | | <0.01 | |  | 1.87 | | 0.08 | |  | 29 | | <0.01 | |  | -3.30 | | <0.01 | |  | 1.29 | | 0.20 | |  | 18.21 | <0.01 | 2 |
| **Light** | | | | | | | | | | | | | | | | | | | | | | | | | | | | | |
|  | *A. aquaticus* | -1.69 | | 0.11 | |  | -1.68 | | 0.11 | |  | 220 | | 0.60 | |  | -1.92 | | 0.06 | |  | -0.54 | | 0.59 | |  | 3.06 | 0.2 | 2 |
|  | *G. pulex* | -0.62 | | 0.55 | |  | 0.55 | | 0.59 | |  | 72 | | 0.71 | |  | -1.18 | | 0.26 | |  | -1.04 | | 0.33 | |  | 3.72 | 0.16 | 2 |
| **Density** | | | | | | | | | | | | | | | | | | | | | | | | | | | | | |
|  |  | df | F | | p |  | df | F | | p |  | df | Χ^2^ | | p |  | df | F | | p |  | df | F | | p |  | W | p | df |
|  | *A. aquaticus* | 41.47 | 2.21 | | 0.11 |  | 40.61 | 0.97 | | 0.42 |  | 3 | 5.47 | | 0.14 |  | 41.18 | 1.73 | | 0.18 |  | 39.85 | 3.37 | | 0.03 |  | 4.98 | 0.55 | 6 |
|  | *G. pulex* | 19.09 | 3.66 | | 0.03 |  | 19.09 | 3.99 | | 0.03 |  | 3 | 10.88 | | 0.01 |  | 22.06 | 5.48 | | 0.01 |  | 19.07 | 0.21 | | 0.89 |  | 17.99 | 0.01 | 6 |
| ^a^ Welch’s t-test for 2-sample comparison  ^b^ ANOVA for multi-sample comparison  ^c^ Wilcoxon’s rank sum test for 2-sample comparison  ^d^ Kruskal-Wallis test for multi-sample comparison  ^e^ Watson-Wheeler test for 2- and multi-sample comparison | | | | | | | | | | | | | | | | | | | | | | | | | | | | | |


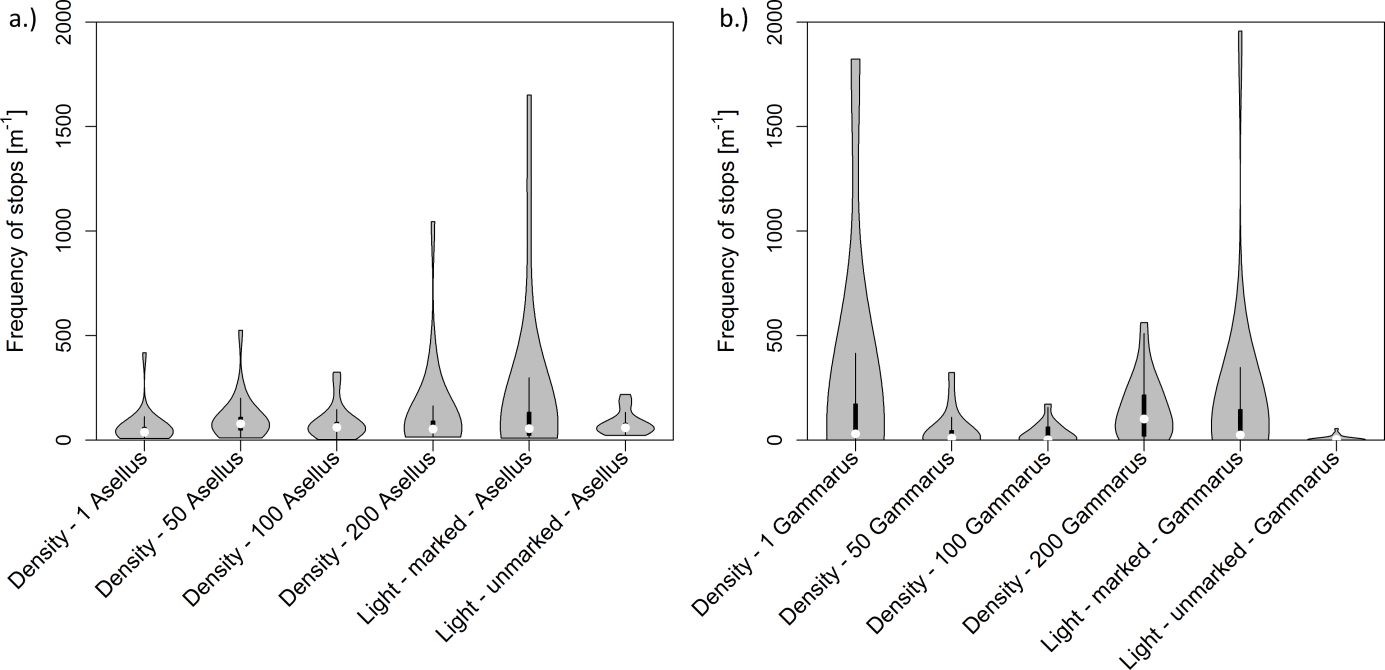


**Figure S2.1:** Violin and boxplots indicating the effects of different testing regimes on the distribution of the number of stops made by individual *A. aquaticus* (a) and *G. pulex* (b) per meter.
